# Spirituality and Prayer on Teacher Stress and Burnout in an Italian Cohort: A Pilot, Before-After Controlled Study

**DOI:** 10.3389/fpsyg.2019.02933

**Published:** 2020-01-21

**Authors:** Francesco Chirico, Manoj Sharma, Salvatore Zaffina, Nicola Magnavita

**Affiliations:** ^1^Post-graduate School in Occupational Health, Catholic University of the Sacred Heart, Rome, Italy; ^2^Department of Behavioral and Environmental Health, School of Public Health, Jackson State University, Jackson, MS, United States; ^3^School of Health Sciences, Walden University, Minneapolis, MN, United States; ^4^Health for All, Omaha, NE, United States; ^5^Occupational Health Service, Pediatric Hospital Bambino Gesù, IRCCS, Rome, Italy; ^6^Department of Woman and Child Health and Public Health, Agostino Gemelli University Policlinic, IRCCS, Rome, Italy

**Keywords:** clinical trial, job burnout, job satisfaction, mental health, meditation, occupational health, prayer, teachers

## Abstract

**Introduction:**

Teaching is a stressful profession that exposes workers to the risk of burnout. Techniques involving higher mental functions, such as transcendental meditation and prayer, have been used in stress and burnout prevention programs. In this study, we report the results of an experience conducted in a group of teachers of a religious institute, in which prayer was used as a technique to prevent burnout.

**Methods:**

Fifty teachers and support staff employed at a Catholic school of a Congregation of nuns volunteered for this study. They were randomized into two groups: prayer treatment (*n* = 25) or control group (*n* = 25). The treatment protocol was based on the combination of individual Christian prayer and a focus group of prayer-reflection. The participants received two 30 min training sessions a week over 2 months. Job satisfaction, well-being, and burnout symptoms (emotional exhaustion and depersonalization sub-scales) were measured at baseline and at follow-up (4 months) with the Italian versions of the Maslach Burnout Inventory validated for teaching and education sector, the General Health Questionnaire, and the Warr, Cook, and Wall’s Job Satisfaction Scale.

**Results:**

At follow-up, a significant improvement of all outcome measures was observed. Emotional exhaustion (16.80–4.92, *p* < 0.001), depersonalization (3.72–0.60, *p* < 0.001) levels, and psychological impairment (10.08–2.04, *p* < 0.001) were significantly decreased, and job satisfaction (45.96–77.00, *p* < 0.001) was increased. The effect sizes (Glass’ Δ) of the therapeutic interventions ranged from 0.53 (satisfaction level) to 2.87 (psychological health), suggesting moderate to large effects.

**Discussion:**

Prayer could be effective, no less than meditation and other spiritual or mind-body techniques, in contrasting the negative effects of occupational stress and preventing burnout among teachers and possibly other human service professionals.

## Introduction

Teaching is a high-stress profession, and many teachers are exposed to high levels of emotional distress at the workplace (Borrelli et al., 2014; Fiorilli et al., 2015; De Stasio et al., 2017; Herman et al., 2018; Chirico et al., 2019b). Teacher stress has been defined as the experience of unpleasant negative emotions such as anger, frustration, anxiety, depression, and nervousness, resulting from some aspect of work (Kyriacou, 2001). Teacher burnout has been defined as a psychological condition that leads to exhaustion, depersonalization, and decreased teacher achievement and self-worth (Evers, 2011). Recently, burnout syndrome (BOS) has been included in the 11th Revision of the International Classification of Diseases as an occupational phenomenon, resulting from chronic workplace stress that has not been successfully managed. BOS is characterized by three dimensions: (1) feelings of energy depletion or exhaustion; (2) increased mental distance from one’s job, or feelings of negativism or cynicism related to one’s job; and (3) reduced professional efficacy (Chirico, 2017b; World Health Organization, 2019). However, BOS and work-related stress-strain should be considered as two different constructs and therefore as two different psychosocial risk factors to be both specifically addressed by employers at the workplace (Chirico, 2015, 2017a,d). According to the British Health and Safety Executive, the consequence of work-related stress is “the adverse reaction people have to excessive pressures or other types of demand placed on them at work” (HSE- Health and Safety Executive, 2019, p. 42). This negative consequence is generally called strain, or distress. In other terms, occupational distress is not an illness, yet if excessive and prolonged, it can result in several physical and mental illnesses including BOS, among others. BOS therefore may be also considered as a consequence of long-term occupational stress-strain associated with negative health outcomes at individual and organizational levels, including mental health issues like anxiety and depression, low job satisfaction, low performance and student care, and high absenteeism and turnover rates (Chirico, 2016a).

Spirituality is a very broad concept, which includes the search for meaning in life and can involve a sense of connection to something greater than ourselves. From a Christian perspective, it has been defined as a way a person lives his/her everyday life in view of his/her relationship to the gods/the “spirit world” and can be considered as that which animates a person’s life of faith and moves a person’s faith to greater depths and perfection (McGrawth, 1999). In the literature, there are also other definitions, that depend on the different opinions of the researchers. Despite the methodological difficulties arising from the different constructs and definitions of spirituality (Martsolf and Mickley, 1998; Buck, 2006; Weathers et al., 2016), there is agreement that the concept of spirituality is not limited to religiosity, and that it includes all forms of meditation (Steinhorn et al., 2017) and mindfulness. Meditation has long been proposed as a prevention strategy for stress-strain and BOS. In the literature, there are several systematic reviews on the efficacy of meditative interventions to reduce physician burnout, yet with inconsistent findings. Indeed, in a systematic review of randomized clinical trials by Dharmawardene et al. (2016), meditative interventions provided a small to moderate benefit for informal caregivers and health professionals for stress reduction, but more research had been advocated by authors to establish effects on burnout. Conversely, a systematic research review by Busireddy reported decreases in depersonalization scores and reduced emotional exhaustion, through self-care workshops and meditation interventions, respectively (Busireddy et al., 2017). In a 4-month study intervention, Elder et al. (2014) showed that the Transcendental Meditation program was effective in reducing psychological distress and burnout even in teachers and support staff working employed at a therapeutic school for students with behavioral problems. Mindfulness can be defined as “the awareness that emerges through paying attention, on purpose, and non-judgmentally to the unfolding of experience moment by moment” (Kabat-Zinn, 2003, p. 145). There is strong evidence that mindfulness practice can reduce job burnout among health care professionals and teachers (Luken and Sammons, 2016).

Religiousness is a particular form of spirituality that proved to be effective in patients with cardiovascular disease or cancer and their caregivers (Movafagh et al., 2017; Abu et al., 2018; Faccio et al., 2018), as well as in depression and mental health problems (Weber and Pargament, 2014; AbdAleati et al., 2016; Braam and Koenig, 2019). In the literature, there are also some studies showing the effectiveness of prayer to tackle occupational stress and burnout. The prayer has been considered as a powerful spiritual coping mechanism in illness (Baldacchino and Draper, 2001), and perception of the transcendent was reported to have a positive influence for stress-related impairment of health in German pastorals (Frick et al., 2016). However, few empirical studies have been conducted on the health effects of Judeo-Christian contemplative prayer practices (Ferguson et al., 2010), especially as a means of coping to address occupational stress and burnout. A research study has documented the use of prayer as an effective coping strategy against work-related stress in a sample of 916 Christian educators, showing a significant relationship between frequency of prayer and job satisfaction (LaBarbera and Hetzel, 2016), and a review showed the essential role of spiritual intelligence in job burnout among teachers (Mirshahi and Barani, 2016). However, although scholars believe that prayer is a valuable tool to be used in nursing and medicine, there is still much controversy on this tool (Sciarra, 2013). Indeed, it is difficult to have a full understanding and unique definition of prayer. Paloma and Pendleton identified four different types of prayer: (i) petitionary prayer, that is, specific requests for oneself or others; (ii) colloquial prayer, that is, a conversational style of prayer in which people may ask for personal guidance, forgiveness, or general blessings; (iii) ritual prayer, that is, memorized prayers or prayers from books; and (iv) meditative prayer, that is, prayer involving reflection upon, and adoration of the divine. According to the authors, each of the well-being measures considered in their studies was influenced by only one type of prayer (Paloma and Pendleton, 1991). According to Jors et al. (2015), prayer could be defined in many ways, based on individual beliefs and religious traditions, but personal prayer involves “the raising up of one’s mind to God” (Damascene, 1864), whereas personal meditation is often considered as a mental exercise or state of being involving reflection or contemplation or mindfulness without being directed toward a higher being (Jors et al., 2015).

The literature on this type of interventions is very scarce, and to our knowledge, there are neither systematic reviews nor randomized controlled trials to support its use among teachers. Therefore, in this study, we wanted to conduct a BOS prevention intervention in the teachers of a religious institute and verify the results of the intervention on the psychological quantities associated with BOS, job satisfaction, and psychological well-being.

## Materials and Methods

### Participants and Setting

Workers from a Catholic school of a Congregation of nuns were invited to participate in a workplace health promotion campaign. This population was chosen because the workers had been selected by the employer based on their religious beliefs and adherence to religious practices. They all had experience of Catholic prayer. Under Italian law, all workers were subjected to health surveillance in the workplace, due to the fact that they were occupationally exposed to biological risk or other occupational risks. The promotion program was conducted by the occupational doctor in charge of their surveillance (FC), who had access to their personal medical records. The study population consisted of teachers: (i) working in the institution for over 6 months with a minimum of a 20-h-per-week; (ii) being a lay teacher employed at a primary, preschool, or kindergarten school of the Congregation; (iii) having no abuse of alcohol or drugs consumption with neurological effects; (iv) having no history of psychiatric disorders; and (v) being not unfit for work. Since 62 out of 66 teachers were females, males were excluded from the study.

From among 62 potentially eligible employees, 50 teachers volunteered for this study. Employees were randomized to two groups: prayer treatment (PT) (*n* = 25) and control group (*n* = 25). Recruitment, intervention, and follow-up took place between September 2017 and February 2018. The study was conducted according to the Declaration of Helsinki. Written and verbal informed consent was obtained from each participant following verbal description of all experimental details, with this obtained priority to any experimental data collection.

### Interventions and Randomization

In a Christian prospective, prayer can take different forms, among which are conversational prayer, meditative prayer, ritual prayer, and intercessory prayer. In this study, we used meditative prayer, which consists of contemplation of spiritual themes and the relationship of the divine with the mankind (Jantos and Kiat, 2007).

Intervention protocol consisted of 16 training sessions that occurred over 8 consecutive weeks, with a maximum of two training sessions per week and a minimum of 1-day rest between training sessions. The participants received two ∼30 min training sessions a week for 2 months before the conclusion of the experiment, delivered by the same expert in religion psychology for all participants. The protocol required the combination of an individualized Christian prayer and a focus group of prayer-reflection. In this study, we used for the intervention group a combination of ritual Christian prayers with the meditative adoration of the divine.

Before the treatment, participants attended two didactic lectures, followed by an individual interview with the instructor. Participants were advised to practice the prayer once daily for 10 min at home before sleeping. Participants in the control group continued with their usual schedule and were not instructed in PT until after the 2-month intervention study was concluded.

Simple randomization procedures were used to assign participants to groups. The schedule of treatment group allocations was concealed by the study statistician, with individual treatment group assignments revealed to the project manager only when study participants completed baseline testing and were ready to commence treatment. All measures were drawn from self-administered questionnaires, to avoid any interviewer bias.

### Outcome Measures

Participants were administered a battery of tests at baseline, before instruction in the PT program. The baseline testing took place at the end of September 2017. Participants were then administered the same battery of tests approximately 4 months later, at the beginning of February 2018. The primary outcomes measures were job satisfaction, psychological well-being, and burnout-related variables (emotional exhaustion and depersonalization). According to the authors who first described the syndrome, burnout is not defined by the sum of the three components, and the third, reduced efficacy, correlates scarcely with the other two (Leiter and Maslach, 2016); consequently, many researchers have chosen to measure the first two components, emotional exhaustion and depersonalization, or only one of the two (West et al., 2009). Consequently, we measured EE and DP.

For the assessment of BOS, the Italian version of the Maslach Burnout Inventory (MBI) validated for teaching and education sector (Sirigatti and Stafanile, 1993) was used. Participants were asked to rate from 0 (never) to 6 (daily) how often they experienced feelings described in each of the 14 items. The questionnaire consisted of the two “core” dimensions of BOS: emotional exhaustion (nine items), e.g., “I feel frustrated by my job,” and depersonalization (five items), e.g., “I don’t really care what happens to some students.” Emotional exhaustion (EE) values ranged from 0 to 54, whereas depersonalization (DP) values ranged from 0 to 30. Cronbach’s α coefficient was 0.85 for exhaustion and 0.77 for depersonalization.

Psychological status was evaluated using the General Health Questionnaire (Goldberg and Williams, 1988) in its version consisting of 30 items (Lattanzi et al., 1988). The General Health Questionnaire scoring traditional method entails that each item is scored 0 for the answers “much worse than usual” or “worse than usual,” and 1 for the answers “better than usual” or “same as usual.” The sum of each item score gives an overall total score (range, 0–30). The score obtained is indicative of the level of psychological malaise. Cronbach’s α in this study was 0.81.

Job satisfaction level was obtained by using the Warr, Cook, and Wall’s Job Satisfaction Scale (Warr et al., 1979), in its Italian version (Cronbach’s alpha = 0.94) (Magnavita et al., 2007). The questionnaire is a 7-point Likert scale composed of 15 items (score ranges, 15–105), which gives a total score, indicating the job satisfaction level. Cronbach’s α in this study was 0.84.

Compliance with the home practice of PT was measured by each participant’s self-report at post-testing.

### Statistical Analysis

Descriptive analyses were conducted for demographic variables (age groupings, marital status, religious belief, teaching class, length of service), and differences between PT and control group were evaluated using the chi-square test. Unpaired and paired *t*-tests were used for comparisons. The effect size of the treatment, that is, whether the results are clinically relevant or not, was calculated using Cohen’s *D*. The data provided by participants were statistically analyzed using the data-analysis software R, package Rcmdr version 2.5-1 (Fox, 2017).

## Results

Table 1 shows the baseline data of participants. Mean ages were 35.56 (SD 6.8) years in the intervention and 37.50 (8.23) years in the control group, respectively. At baseline, differences between groups in age, educational background, religious belief, teaching class, marital status, and years of experience were not statistically significant.

**TABLE 1:** Baseline data.

	**Prayer intervention group (mean ± SD)**	**Control group (mean ± SD)**	***p* (Student’s *t* test)**
Job satisfaction	45.96 ± 7.64	46.80 ± 8.25	0.711
Psychological health impairment	10.08 ± 3.39	9.44 ± 3.67	0.525
Emotional exhaustion	16.80 ± 9.67	19.44 ± 10.45	0.359
Depersonalization	3.72 ± 2.70	4.16 ± 3.26	0.606

The analysis of the results at the end of the follow-up is shown in Table 2. In the prayer group, a statistically significant improvement of all outcome measures was observed. Emotional exhaustion level decreased from 16.80 to 4.92 (*p* < 0.001); depersonalization, from 3.72 to 0.60 (*p* < 0.001); and psychological impairment, from 10.08 to 2.04 (*p* < 0.001), whereas job satisfaction level increased from 45.96 to 77.00 (*p* < 0.001). The effect sizes (Glass’ Δ) of the therapeutic intervention ranged from 0.53 (for job satisfaction) to 2.87 (for psychological health), suggesting moderate to huge effects. The compliance with the practice of the prayer technique was high; 100% of the participants prayed at least once a day at home. Participants reported no unexpected, study-related serious adverse events.

**TABLE 2:** Four-month change scores for perceived satisfaction, mental health, and teacher burnout.

**Variable**	**Prayer intervention group (eman ± SD)**	**Control group (mean ± SD)**	***p*-value^∗^**	**Effect size^∗∗^**
Satisfaction level	77.00 ± 8.19	46.04 ± 7.50	*p* < 0.001	0.53
Psychological health impairment	2.04 ± 1.39	9.16 ± 3.21	*p* < 0.001	2.87
Emotional exhaustion (EE)	4.92 ± 4.48	19.76 ± 10.86	*p* < 0.001	1.78
Depersonalization (DP)	0.60 ± 0.76	4.24 ± 2.60	*p* < 0.001	1.90

*^∗^Unpaired *t*-test between groups and paired *t*-test within treatment group. ^∗∗^Cohen’s *D*.*

## Discussion

In this experience, we observed a significant increase in job satisfaction and mental well-being, as well as a reduction in the levels of depersonalization and emotional exhaustion, in the prayer group, if compared with the control. It is important to note that the two groups shared the same religious belief and were both used to Christian prayer. The results of this research indicate that the prayer training experiment, which was developed in the framework of a voluntary workplace health program, was effective in BOS prevention in a sample of female lay teachers employed in a Catholic school. At the end of the intervention period, workers of the prayer group reported significant improvements compared to controls for all examined outcome variables, i.e., job satisfaction, mental well-being, and emotional exhaustion and depersonalizations that are considered in the literature as the “core” dimensions of BOS. The intervention could be considered of moderate to large effect size, with the largest effect measured on psychological stress and subscales of BOS.

The results of this study are not unexpected, based on the literature. Prayer, indeed, has been considered a Western form of transcendental meditation, leading to a psychological and physical status of well-being (Jantos and Kiat, 2007). Numerous studies have shown that Christian and non-Christian prayer can have beneficial effects. An Indian research study revealed that workplace spirituality moderates the negative relationship of stress and health, and positively correlates with health (Kumar and Kumar, 2014). Benevene and Fiorilli (2015) comparing burnout of Catholic consecrated teachers with lay teachers from Italian public and Catholic schools pointed out that teachers from public schools obtained higher means on the emotional exhaustion and depersonalization dimensions (Benevene and Fiorilli, 2015). Zhang et al. (2019) showed that enhancing the spiritual climate among nurses can enhance job satisfaction and reduce nursing burnout and turnover intention. Chandler (2010) has underlined the role that prayer time plays in preventing burnout in ministry. Our study is also in agreement with findings of past research showing how religiousness is associated with low levels of burnout in helping professions like medical students (Wachholtz and Rogoff, 2013), end-of-life care setting operators (Holland and Neimeyer, 2005), hospital employees (Chirico et al., 2018; Carneiro et al., 2019), nurses (Kovacs and Kèdzy, 2008; Chirico et al., 2018), nuns serving as nurses (Kovacs, 2009), and lay and consecrated school teachers (Benevene and Callea, 2011; Chirico, 2017c). According to Frederick, Christian human services workers who practice three spiritual exercises, i.e., the Jesus Prayer, the daily examination, and the prayer of consideration, by reconnecting with the empowering, living spirit of God, may effectively prevent and cope with burnout (Frederick et al., 2017). In past experimental studies, the impact of group sessions and individual practice of Centering Prayer two times daily was hypothesized to decrease stress and increase “collaborative relationship with God” in Catholic congregants (Pargament et al., 1988; Ferguson et al., 2010).

Not only prayer but also other forms of Meditation produce desirable physiological changes, by means of the so-called “relaxation response” (Benson, 1975). In the literature, there are many studies about the effectiveness of mindfulness techniques and training programs to alleviate and reduce job burnout in nurses and health professionals (Smith, 2014; White, 2014). They have been found to increase personal presence and empathy and combat stress, burnout, and anxiety among human service professionals as well (McCollum, 2015). Other research showed that higher levels of spirituality reduce levels of burnout among individuals (Captari, 2010) and dimensions of spirituality can buffer the negative effects of burnout (Amen, 2006; Bade and Cook, 2008; Bänziger et al., 2008). For instance, Frederick describes as mindfulness, differentiation of self, and Christian spiritual practices may play a role in preventing and coping with burnout (Frederick et al., 2017). Based on all these studies, spirituality has been considered as a useful coping strategy to address BOS (Doolittle et al., 2013), to be included in the WHO’s health definition with a holistic view of this concept (Chirico, 2016b; Chirico and Magnavita, 2019).

Our study confirms also that the results of a quasi-experimental (pre/posttest) pilot study showing a significant improvement of mental health outcomes occurred in a convenience sample of 27 teachers and professional staff working in special education with the use of mindfulness and prayer (Sharp Donahoo et al., 2018). Both meditation and prayer are mind-body-spirit interventions and just differ in the conditioning of the mind. If one is conditioned from birth to believe in the external form of support, prayer seems to be effective, while if someone is conditioned from birth to believe in the internal form of support, meditation seems to be beneficial (Sharma, 2018). Past studies showed that the practice of meditation can lower stress-strain levels by a decreased sympathetic nervous system and hypothalamic-pituitary-adrenal axis and reductions in elevated cortisol (stress hormone) levels (Walton et al., 2004). Therefore, based on our findings, the “meditative” prayer would have the potential to do likewise. In the literature, prayers, meditation, or similar rituals like yoga have all been considered as emotion-focused coping strategies capable of reducing and managing the negative and distress emotions experienced by healthcare professionals at the workplace (Giacalone and Jurkiewicz, 2003). However, despite a growing body of research connecting mindfulness to Christian spiritual practices (Frederick et al., 2017) and emerging evidence of the importance of these spiritual practices when used as coping against stress, worry, and burnout, they are underestimated.

Strengths of this work were a randomized control design, with the inclusion of all study participants employed at schools of the same Congregation. The health surveillance carried out at the workplace has prompted the full participation of teachers who were all post-tested. Furthermore, this was one of the few studies focusing on the beneficial effects of prayer on mental health and burnout levels of teachers. Study limitations included reduced generalizability of a study conducted in a single private school to other types of school or other types of religion. Other studies, conducted on subjects of other religions and which include people not proficient in prayer, may be useful to deepen the results. Furthermore, participants could not be blinded to their treatment assignment. Finally, the use of self-report instruments to measure outcome variables introduced the possibility of bias. However, this was an original pilot study that by identifying a new key area requires further research in this field to analyze the beneficial effects of prayer in other fields and professions.

### Practical Implications

This research has important implications because it is one of the few studies showing that prayer could be effective, especially in religious contexts, no less than meditation and other spiritual or mind-body techniques, to contrast the negative effects of stress-strain and burnout at workplace among teachers (Luken and Sammons, 2016). Practical implications of the research refer to the usage of prayer alternatively or in common with meditation in the framework of workplace health programs, to improve mental well-being, especially in occupational settings like helping professions and human service professionals in which risks arising from work-related stress and occupational burnout hazards can be especially important. In the European Union, interventions to address all the psychosocial hazards at the workplace are mandatory for all employers (Chirico et al., 2019a). However, evidence-based prevention and control strategies in the public health field should be evaluated in terms of cost-effectiveness. The role of religion and spirituality in the promotion of health has been discussed in numerous medical publications (Kirschner, 2003). Workplace health promotion may give several benefits to employees and organizations, due to a safe and healthy work environment (WHO).

## Conclusion

Our study therefore confirms that spirituality could be used in the framework of workplace health promotion programs to improve employees’ performances and organizational effectiveness, especially in professions, e.g., the teaching, where emotional demands are very high (Karakas, 2010). Prayer and other spiritual techniques could increase the individual resources of workers. As shown by Demerouti et al. (2001) in the Job-demand resources model, more spirituality-related individual resources may buffer the negative effect of job demands on job strain, including burnout, and influence motivation, by promoting the work engagement, when job demands are high.

## Data Availability Statement

The datasets generated for this study are available on request to the corresponding author.

## Ethics Statement

Ethical review and approval was not required for the study on human participants in accordance with the local legislation and institutional requirements. The patients/participants provided their written informed consent to participate in this study.

## Author Contributions

FC drafted the manuscript. NM and MS revised the manuscript. FC and SZ performed statistical analyses.

## Conflict of Interest

The authors declare that the research was conducted in the absence of any commercial or financial relationships that could be construed as a potential conflict of interest.
